# Brood parasitism and egg recognition in three bunting hosts of the cuckoos

**DOI:** 10.1002/ece3.10659

**Published:** 2023-10-20

**Authors:** Yuhan Zhang, Guo Zhong, Guixia Wan, Longwu Wang, Wei Liang

**Affiliations:** ^1^ Ministry of Education Key Laboratory for Ecology of Tropical Islands, College of Life Sciences Hainan Normal University Haikou China; ^2^ School of Life Sciences Guizhou Normal University Guiyang China

**Keywords:** arms race, brood parasitism, bunting, common cuckoo, egg recognition, ground‐nesting birds

## Abstract

Comparative studies of egg recognition and rejection between various sympatric hosts provide insight into the coevolutionary history of the hosts and parasites, as well as the degree of antagonism between the species. Although buntings are widely considered to be a suitable host taxon for cuckoos, there has been relatively little research on this example of parasitism and host antiparasitic behaviour. Here we provided the first report on brood parasitism and egg recognition in three sympatric ground‐nesting bunting hosts of the common cuckoo (*Cuculus canorus*), namely the yellow‐throated bunting (*Emberiza elegans*), south rock bunting (*E. yunnanensis*), and crested bunting (*E. lathami*). The results show that for the five breeding seasons during 2018–2022, the parasitism rate by common cuckoos was 0.87% and 0.45% in yellow‐throated buntings and south rock buntings, respectively, whereas the parasitism rate by an unidentified parasite was 4% during 2018–2023 in the crested bunting. The rejection rates of the three bunting hosts for blue non‐mimetic eggs were 89.3%, 88.9%, and 100% for yellow‐throated buntings, south rock buntings, and crested buntings, respectively. The rejection rates for red non‐mimetic eggs by yellow‐throated buntings and south rock buntings were lower at 76.9% and 82.4%, respectively. All three sympatric bunting hosts examined had high levels of egg recognition and egg rejection, suggesting that it may have been subjected to high parasitic history and that egg recognition ability was retained after the loss of parasitism, which needs to be further verified by future experiments.

## Graphical abstract

We provided the first report on brood parasitism and egg recognition in three sympatric ground‐nesting bunting hosts of the common cuckoo (*Cuculus canorus*), namely the yellow‐throated bunting (*Emberiza elegans*), south rock bunting (*Emberiza yunnanensis*), and crested bunting (*Emberiza lathami*). The results show that for the five breeding seasons during 2018–2022, the parasitism rate by common cuckoos was 0.87% and 0.45% in yellow‐throated buntings and south rock buntings, respectively, whereas the crested bunting was not found to be parasitised. All three sympatric bunting hosts examined had high levels of egg recognition and egg rejection, suggesting a long history of coevolution with cuckoos.

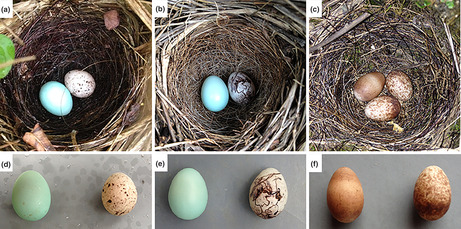

## 1 INTRODUCTION

Brood parasitism can be costly to the host in terms of their reproductive success. Therefore, hosts have evolved a wide range of antiparasitic strategies against parasites under selection pressure to improve their fitness. Hosts and parasites have coevolved adaptations and counter‐adaptations (Davies, 2000; Soler, 2017), such as choosing hidden nest sites, attacking parasites, and recognising and rejecting foreign eggs or parasitic chicks. Of these antiparasitic strategies, one of the most successful has been host recognition and successful rejection of foreign parasitic eggs. (Antonov et al., 2011; Soler et al., 2017; Wang et al., 2020). Brood parasitism is considered an important selective pressure for the evolution of host egg recognition (Røskaft et al., 2002). Some birds have acquired the ability to recognise foreign eggs through experience and/or genetics (Lotem et al., 1992, 1995; Moksnes, 1992; Molina‐Morales et al., 2014; Moskát & Hauber, 2007). Generally, the longer the parasitic history or higher the parasitic pressure, the greater the egg recognition ability of the host population and higher the probability of rejecting the foreign eggs (Davies & Brooke, 1988; Kelly, 1987; Soler, 2017). Therefore, populations lacking a parasitic history have a little to no egg recognition ability (Davies & Brooke, 1989a, 1989b; Moksnes et al., 1991). As host egg recognition increased over time, cuckoos (Cuculidae) have also evolved mimetic eggs that more readily matched the host egg phenotypes (Davies, 2000; Feeney et al., 2014; Soler, 2017). As a result, cuckoos diverged into different gentes (Brooke & Davies, 1988; Stoddard & Stevens, 2010, 2011). Therefore, the ability of the host to reject eggs and the mimicry accuracy of the cuckoo eggs can reflect their coevolutionary history within the avian parasitism system.

The antagonistic coevolution between cuckoos and their hosts is a result of the long‐term interaction. However, comparative studies at various spatial and temporal scales have found that antagonistic coevolutionary relationships within different parasitic systems or involved populations can be complex (Soler, 2017). Theoretically, long‐term comparative studies of single parasitic systems over long periods of time could provide direct insight into the coevolutionary processes that occur. Soler et al. (1994) showed that Eurasian magpies (*Pica pica*) increased egg rejection of great spotted cuckoos (*Clamator glandarius*) from 0% to 10% over a 10‐year period in Spain. However, evolutionary time scales pose a significant barrier to such studies. For example, both the parasitism rate of the common cuckoo (*Cuculus canorus*) and the rejection of cuckoo eggs by the host great reed warbler (*Acrocephalus arundinaceus*) remained essentially unchanged over the 70‐year period (Moskát et al., 2008; Zölei et al., 2015). In contrast, comparative studies between different parasitic systems or populations (e.g. Trnka et al., 2023) provide an indirect yet ideal way to reveal the antagonistic coevolutionary relationship between the parasite and host species. For example, Yi et al. (2020) compared the egg recognition ability of different hosts breeding in the same area to explore the correlation between egg rejection and cuckoo parasitism. However, given that there are many species of passerine birds suitable for cuckoo parasitism breeding within the same area, only a very few species are regularly parasitised by cuckoos, and there are few studies on why these potential hosts are not, or seldom, parasitised (Davies, 2000; Grim et al., 2011; Grim & Honza, 2001; Honza et al., 2004).

Buntings are small finch‐like birds in the family Emberizidae that generally nest in open, bowl‐shaped nests on the ground and have large populations, feeding mostly on insects and certain grains (Ding et al., 2019). Species with these characteristics are generally considered to be suitable hosts for cuckoos (Soler et al., 1999). As of now, the only recorded bunting species that act as hosts for the cuckoo in China are the black‐faced bunting (*Emberiza spodocephala*), yellow‐throated bunting (*E. elegans*), and Jankowski's bunting (*E. jankowskii*) (Yang et al., 2012). Studies on the suitability of the yellowhammer (*E. citrinella*) and common reed (*E. schoeniclus*) buntings as hosts for cuckoos and on their egg recognition ability have been previously reported (Moksnes & Røskaft, 1995; Wyllie, 1981). Both host species have strong egg recognition abilities (Davies & Brooke, 1989a; Procházka & Honza, 2004). However, Antonov et al. (2006) investigated the parasitism and egg recognition of the corn bunting (*E. calandra*) and found that it had a higher parasitism rate than that of other buntings and only a moderate rejection rate (46%) of non‐mimetic eggs, suggesting they are suitable hosts for cuckoos.

**FIGURE 1:**
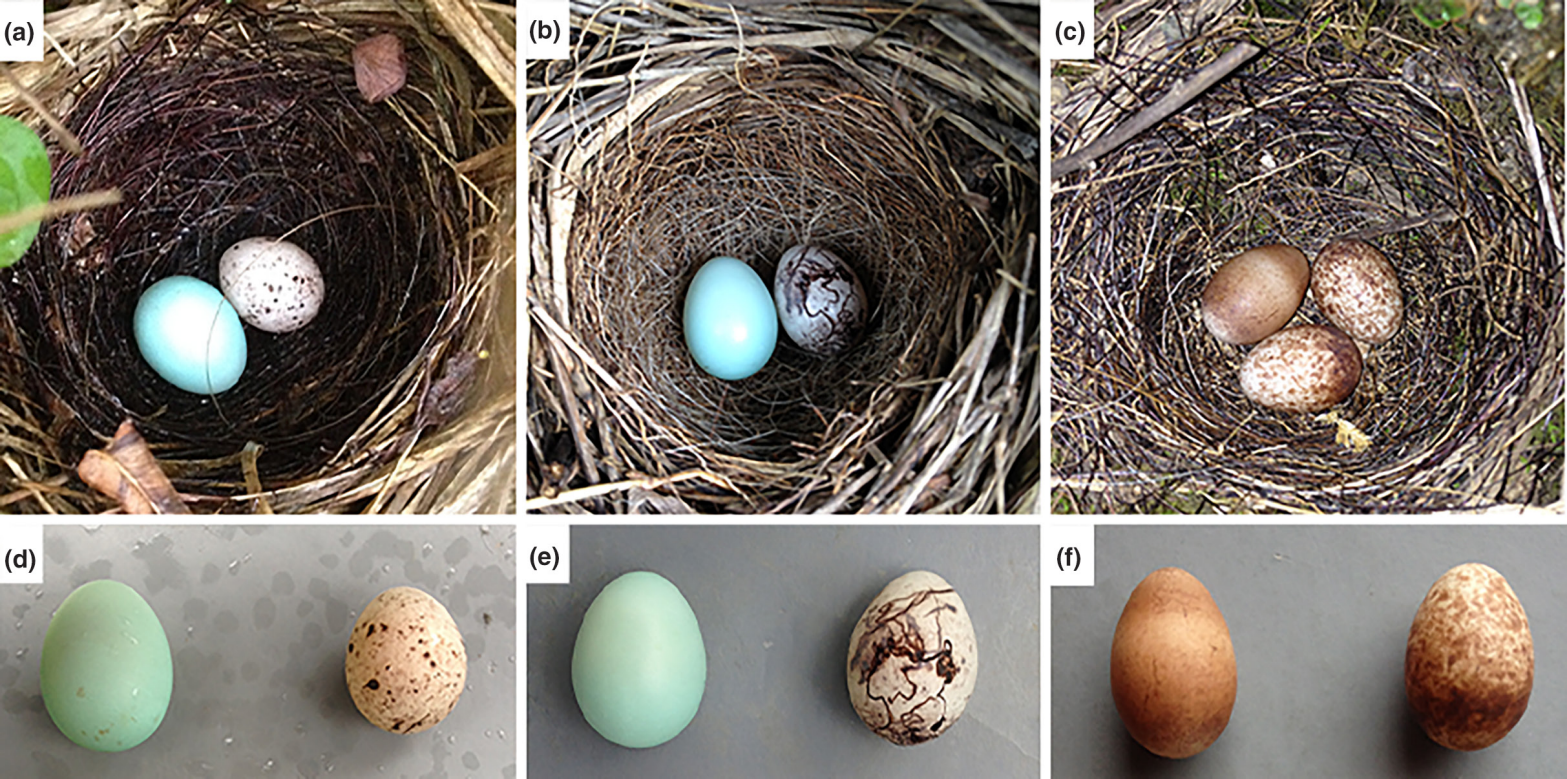
Photos of three bunting hosts' nests and parasitic egg comparisons. (a,d) showing the yellow‐throated bunting nest parasitised by the common cuckoo, (b,e) showing the south rock bunting nest parasitised by the common cuckoo, (c,f) showing the crested bunting nest parasitised by an unidentified parasite.

In this study, the yellow‐throated bunting, south rock bunting (*Emberiza yunnanensis*), and crested bunting (*E. lathami*) were bred within the same area. All examined species were ground nesters and had similar open, bowl‐shaped nests. Past studies have reported parasitism of the yellow‐throated bunting by both the Himalayan cuckoo (*C. saturatus*) and Sunda cuckoo (*C. lepidus*) (Lowther, 2019; Yang et al., 2012), as well as parasitism of the crested bunting by the common cuckoo (Lowther, 2019). No cases of parasitism of the south rock bunting have been recorded. In this study, we investigated the parasitism of these three species of buntings by the common cuckoo in the same area. The main common cuckoo population in the study area uses the grey bushchat (*Saxicola ferreus*) as its primary host, laying eggs of a blue phenotype (Zhong, 2020), which does not mimic the eggs of the three species of buntings. Model eggs have been used in many previous studies (e.g. Grim et al., 2011; Samaš et al., 2014; Yang, Wang, et al., 2014), especially for comparing the behaviour of hosts from different species and populations (Hauber et al., 2015). As it has been reported that some hosts are more receptive to red foreign eggs than to other colours (Higuchi, 1989; Liu, Wang, & Liang, 2023), we therefore tested the egg recognition ability of the three host species by placing non‐mimetic blue and red model eggs simulating cuckoo eggs in each of their nests. We predicted that all the three bunting species would have strong egg recognition ability and be able to identify foreign eggs that are different from their own ones, while they would have lower egg rejection with red experimental eggs than those with blue ones (Liu, Wang, & Liang, 2023). Based on this, we further predicted that the egg colour of the cuckoo should accurately mimic the egg colour of its target hosts (Figure 1).

## 2 MATERIALS AND METHODS

### 2.1 Study area and species

The field study site is located in Liuzhi (26°10′–26°14′ N, 105°13′–105°24′ E), Guizhou, southwestern China, at an altitude of 1287–1657 m, which is a subtropical monsoon climate area. The study area feature is a karst mountainous landscape, consisting of villages, arable lands, rivers, shrubs, and barren slopes (Liu, Zhang, et al., 2023).

The yellow‐throated bunting is found across China (Zheng, 2017), and lays clutches of 3–6 eggs. Their eggs are characterised as greyish white in colour with brown spots (Chen et al., 2015). The crested bunting is primarily found in Tibet, Yunnan, Sichuan, Guizhou, and Hunan in southern China (Zheng, 2017), and generally lays clutches of 3–5 eggs. Their eggs are characterised as greyish white in colour with reddish brown or purplish brown spots, primarily concentrated at the blunt end of the egg (Zhao., 2001). Godlewski's bunting (*E. godlewskii*) generally has a clutch size of 3–5 eggs. Their eggs are characterised as greyish white in colour with black line markings (Ding et al., 2019). Comparison of egg phenotypes of the three species of buntings with those of common cuckoos is shown in figure 1. The south rock bunting was previously considered to be a subspecies of Godlewski's bunting, and has recently been identified as a separate species (Li et al., 2023). For the five breeding seasons (April–August) during 2018–2022, these three bird species all bred in the study area as ground breeders with open, bowl‐shaped nests.

### 2.2 Searching for experimental nests

Nests of all three species of buntings were located on the ground or on low branches of vegetation, usually hidden under thickets or grasses, and also nested on soil ridges along cornfields and between rock crevices. Thorough searches for experimental nests were conducted from April to August in the study area, incorporating nest site preferences of these three species of buntings.

### 2.3 Measurement of cuckoo parasitism and eggs

Each experimental nest was numbered, and their GPS locations were recorded. The presence or absence of parasitism was regularly checked and recorded. The eggs were weighed using an electronic jewellery scale (measurement range 500 g, accuracy to 0.01 g). The dimensions of eggs of the three species as well as the two types of model eggs were measured using digital callipers (GB/T1214.2_1996, accuracy to 0.02 mm), and the egg volumes were calculated (Hoyt, 1979). Only one egg from each experimental nest was taken for measurement.

### 2.4 Experiments examining egg recognition ability

Two sets of experiments were performed to evaluate the egg recognition abilities of the three host species: the red model egg group and the blue model egg group. Clay was used to make both the red and blue model eggs. The egg mass and sizes are shown in Table 1. Either one red or one blue model egg was placed in an experimental nest before mid‐incubation (Yi et al., 2020). Nest checks were conducted every 3 days for a 6‐day experimental cycle, and the results of the parent's response to the foreign egg were recorded (Yi et al., 2020). If the model egg was pecked by the parent, thrown out of the nest, or if the nest was abandoned, the host response was recorded as rejection. If the model egg was intact and the host continued to incubate the egg, the host response was recorded as acceptance. The experiment was not repeated multiple times within the same nest (Yang, Su, et al., 2015). The sample size for each set of experiments was above 15.

**TABLE 1:** Comparison of parameters of bunting host eggs, parasitic eggs, and non‐mimetic model eggs used in this study.

Egg type	Experimental species	*N*=	Egg mass (g)	Egg length (mm)	Egg width (mm)	Egg volume (mm^3^)	Sources
Host	*Emberiza elegans*	15	2.08 ± 0.18	19.15 ± 0.87	15.01 ± 0.29	2.20 ± 0.15	This study
Host	*E. yunnanensis*	27	2.61 ± 0.25	20.58 ± 0.83	15.62 ± 0.50	2.57 ± 0.22	This study
Host	*E. lathami*			17.9–22.0	13.0–17.0		Zhao (2001)
*Cuculus canorus*	Host *E. elegans*	1	3.21	22.32	17.32	3.41	This study
*Cuculus canorus*	Host *E. yunnanensis*	1	3.26	22.35	17.78	3.60	This study
*Unidentified parasite*	Host *E. lathami*	1		23.14	15.79	2.94	This study
Red model egg	*E. elegans*	20	2.57 ± 0.03	18.94 ± 0.57	14.45 ± 0.31	2.01 ± 0.09	This study
Blue model egg	*E. elegans*	20	2.65 ± 0.09	19.50 ± 0.58	14.89 ± 0.42	2.21 ± 0.16	This study
Red model egg	*E. yunnanensis*	20	3.09 ± 0.34	19.65 ± 0.72	15.38 ± 0.36	2.37 ± 0.11	This study
Blue model egg	*E. yunnanensis* and *E. lathami*	20	3.14 ± 0.03	20.56 ± 0.41	15.84 ± 0.37	2.63 ± 0.11	This study

### 2.5 Statistical analysis

The Chi square test and Fisher exact test were used to test the statistical significance of differences in egg rejection rates referring to mode eggs between the three host species. Differences were considered statistically significant at *p* < .05. Statistical analysis was conducted using IBM SPSS Version 26.0 for Windows (IBM Inc., USA), and the data were presented as mean ± standard deviation (SD).

## 3 RESULTS

### 3.1 Natural parasitism rate of the common cuckoo on bunting host species

During 2018–2022, a total of 115 yellow‐throated bunting nests were found, one of which was parasitised by the common cuckoo, with a parasitism rate of 0.87%. A total of 222 south rock bunting nests were found, of which one nest was parasitised by the common cuckoo, with a parasitism rate of 0.45%. A total of 25 crested bunting nests were found during 2018–2023, with a parasitism rate by an unidentified parasite of 4%.

The parasitic eggs in the nest of the yellow‐throated bunting and the south rock bunting were pale blue, and those of the crested bunting were brown, and the parasitic eggs were all non‐mimic eggs (Figure 1). The parasitised yellow‐throated bunting nest contained one of its own egg and one parasitic egg, and the host had abandoned the nest. The parasitised south rock bunting nest contained one of its own eggs and one parasitic egg, and the nest was depredated 3 days later. The parasitised crested bunting nest contained two of its own eggs and one parasitic egg, the parasitic species was unknown and the host had abandoned the nest when found. A comparison of various parameters of the cuckoo and host eggs is shown in Table 1.

### 3.2 Egg recognition and rejection

**FIGURE 2:**
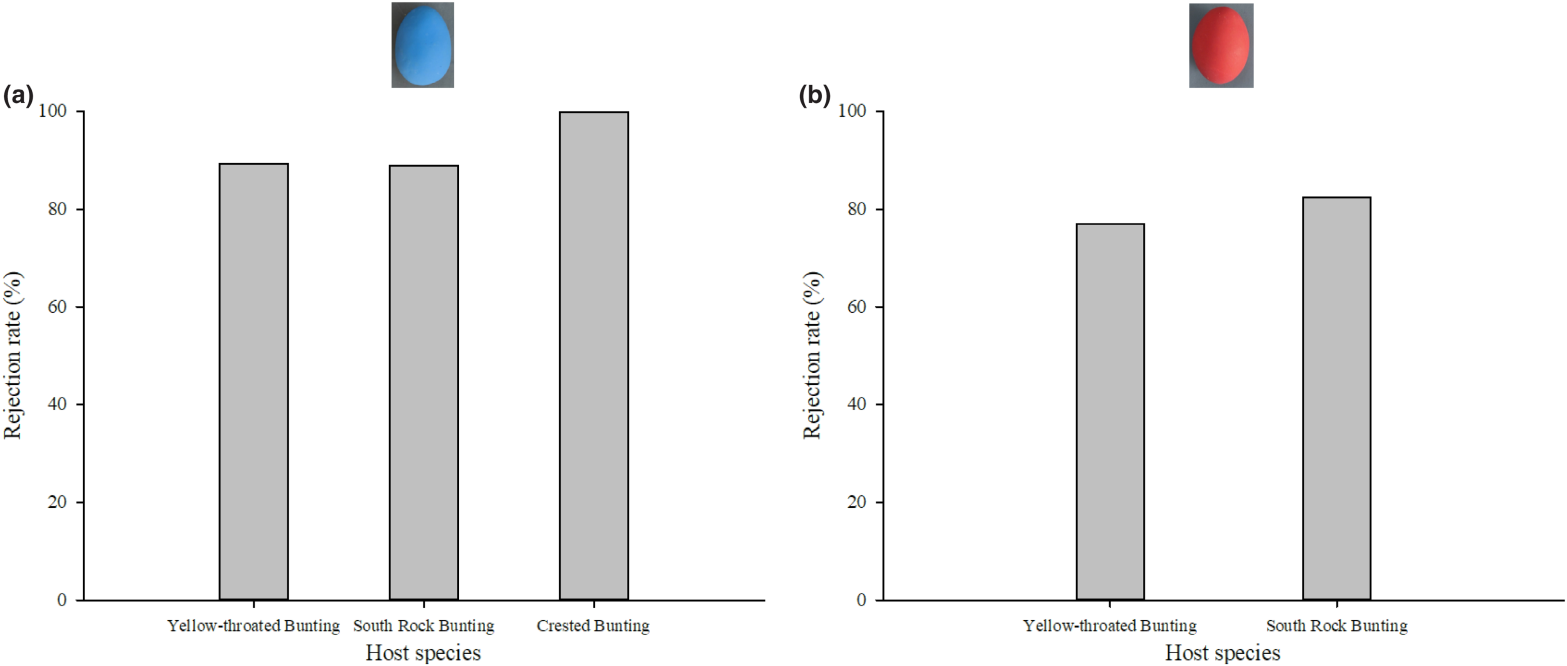
Non‐mimetic egg rejection rate of three bunting hosts. (a) Blue model egg rejection rate of the three bunting hosts, and (b) red model egg rejection rate of the yellow‐throated bunting and south rock bunting.

In the experiment involving blue model eggs, there were a total of 28 yellow‐throated bunting nests, 25 of which were recorded as rejection; a total of 36 south rock bunting nests, 32 of which were recorded as rejection; and 16 crested bunting nests, all of which were recorded as rejection. All three host species were able to identify and reject the blue model eggs in the nest with no significant differences observed in rejection rates (89.3% vs. 88.9% vs. 100%, Chi square tests, χ^2^ = 1.921, *p* = .383; Figure 2).

In the experiment involving red model eggs, there were a total of 13 yellow‐throated bunting nests, 10 of which were recorded as rejection, and a total of 17 south rock bunting nests, 14 of which were recorded as rejection. Both host species were able to identify and reject the red model eggs in the nest with no significant difference observed in rejection rates (76.9% vs. 82.4%, Fisher exact tests, *p* = .100; Figure 2).

Although both yellow‐throated bunting and south rock bunting had higher rejection rates for the blue model eggs than the red model eggs, there was no significant difference (89.3% vs. 76.9%, Fisher exact tests, χ^2^ = 0.322, *p* = .570; 88.9% vs. 82.4%, Fisher exact test, χ^2^ = 0.049, *p* = .825; Figure 2).

## 4 DISCUSSION

Our results show that the cuckoo parasitism rates in all three sympatric bunting host species were low and that the parasitic eggs were all non‐mimetic eggs. The parasitic eggs in the nests of the yellow‐throated bunting and the south rock bunting were solid blue, and the parasitic egg of the crested bunting was brown. All three bunting species showed high recognition and rejection of non‐mimetic foreign eggs, and the yellow‐throated bunting and south rock bunting did not differ significantly in their egg rejection rates for the red and blue model eggs.

Previous studies have shown that the degree of egg rejection by the host of cuckoos was significantly and positively correlated with the degree of parasitism rate (Røskaft et al., 2002), which was not supported by the results of the present study as all three bunting species had strong egg recognition but low cuckoo parasitism rates (for comparison, 9% in corn bunting, *n* = 90, Antonov et al., 2006; 11.5% in Jankowski's bunting, *n* = 95, Zhang et al., 2020). Similarly, the findings of Yi et al. (2020) showed no difference in egg recognition ability between potential hosts and currently used hosts. Since some host species have a strong ability to reject eggs in the absence of parasitism (Lahti, 2006; Medina & Langmore, 2015; Underwood et al., 2004), brood parasitism rates may not be a primary indicator of host egg recognition ability, or that they were once subjected to parasitism pressure and egg recognition ability is retained. Certain host species have little to no egg rejection ability, even under parasitism pressure. There are two main potential explanations for this phenomenon, the evolutionary lag hypothesis, which refers to coevolutionary time being too short for the host to have evolved the ability to recognise eggs (Davies & Brooke, 1989a; Dawkins & Krebs, 1979) and the evolutionary equilibrium hypothesis, which refers to a state of equilibrium in coevolutionary processes, where hosts obtain the highest reproductive benefit (Langmore et al., 2005; Rohwer & Spaw, 1988).

One possible reason for this result was that all three species of buntings have been subjected to high parasitic pressure. Environmental changes may cause birds to lose some previously evolved traits overtime. For example, some hosts have evolved high inter‐clutch egg variation and low intra‐clutch egg variation in defence against brood parasitism during long‐term coevolution. This trait can be gradually lost after the removal of parasitism pressure (Lahti, 2005). Theoretically, hosts also gradually lose their ability to reject eggs when parasitic pressure is removed. For example, Eurasian reed warbler (*Acrocephalus scirpaceus*) populations have shown a decreasing rate of egg rejection as parasitic pressure decreases (Brooke et al., 1998). However, some studies have also shown that if the host escapes the parasitic system, the egg recognition ability of some hosts can be retained for a long time in the absence of parasitism (Honza et al., 2004; Lahti, 2005, 2006; Martín‐Vivaldi et al., 2013; Medina & Langmore, 2015). For example, Yang, Liu, et al. (2014) found that the red‐billed leiothrix (*Leiothrix lutea*), a cuckoo host species introduced to Hawaii 150 years ago, still retains a strong egg rejection ability. All three bunting host species in this study may have been subjected to high levels of parasitic pressure by cuckoos in the past. As a result, they could have evolved a high level of egg recognition over the course of a long‐term coevolution, ultimately winning the ‘arms race’, with egg recognition abilities remaining as a residual behaviour. In contrast, nest sanitation behaviour of parents may also be a factor driving host rejection of foreign eggs (Feng et al., 2019; Li et al., 2021; Yang, Wang, et al., 2015). Nest sanitation behaviour is mainly directed towards irregular, non‐ovoid objects in nests (Guigueno & Sealy, 2009; Hauber et al., 2021; Underwood & Sealy, 2006), which is distinct from egg rejection behaviour. Rothstein (1975) hypothesised that nest sanitation in birds is a pre‐adaptation to egg rejection behaviour. Nest predation is a major factor influencing the reproductive success of birds, as well as their life history and behaviour (Davies, 2000; Lima, 2009; Martin, 1992; Schmidt & Whelan, 1999). Several studies have been suggested that the timely removal of faecal sacs and egg shells from nest can reduce the likelihood of detection by predators (Petit et al., 1989; Tinbergen et al., 1962). Ground‐nesting birds face a higher risk of nest predation compared to other birds (Haskell, 2002; Minias & Janiszewski, 2023; Roos et al., 2018). Therefore, for all three bunting species, timely clearance of phenotype‐specific foreign eggs from the nest likely may have been necessary to reduce the risk of nest predation. However, further studies are needed to determine whether nest sanitation behaviour of the three bunting host species promotes their egg rejection behaviour.

Mimetic eggs are often used in brood parasitism studies to simulate parasitic eggs and test egg recognition in birds. However, studies have found that the model egg size (Roncalli et al., 2017), material (Moksnes et al., 1991; Prather et al., 2007; Roncalli et al., 2017), egg mass (Ruiz‐Raya et al., 2015), and other factors can have an impact on the egg rejection of the host. Higuchi (1989) placed different coloured eggs into nests of the Japanese bush warbler (*Horornis diphone*) and found that the host was significantly more receptive to red eggs than to eggs of other colours. A similar phenomenon was also observed in green‐backed tits (*Parus monticolus*). However, the red model eggs were may be harder to recognise in the darker environment of the cave nests (Liu, Wang, & Liang, 2023). In the present study, the nests of all three buntings are ground‐open nests, and hosts can clearly recognise foreign eggs and no significant difference was observed in egg rejection rates between the blue and red model eggs for the yellow‐throated bunting and south rock bunting, suggesting that egg colour has no effect on its recognition and rejection of foreign eggs in the two bunting hosts.

In this study, the results showed a very low rate of parasitism in all three species of buntings, and one each of yellow‐throated bunting nest and south rock bunting nest were found to be parasitised with pure pale blue eggs that did not match the phenotype of the host eggs. In the study area, the common cuckoo that laid such eggs is known to primarily parasitise the nests of grey bushchats as their primary host (Zhong, 2020). The egg phenotype of the common cuckoo, pale blue eggs, was highly mimetic of that of the grey bushchat (Zhong, 2020). The two parasitic eggs found in this study were of this same phenotype, indicating that they were both laid by common cuckoos that primarily parasitise the nests of grey bushchats. The result that the parasitised egg phenotypes did not mimic the hosts egg phenotypes at all suggests that the three species of buntings did not have a history of coevolution with the common cuckoo of this clade. In contrast, the nests of the yellow‐throated bunting and south rock bunting were parasitised by the common cuckoo for three possible reasons:

1. Possible host transfer. Although the common cuckoo typically parasitises a specific host species, it may parasitise other host species when faced with multiple suitable hosts (Antonov et al., 2010; Yang, Su, et al., 2015). In cases where the primary host has evolved the ability to recognise and reject highly mimetic parasitic eggs, parasites may resort to parasitising alternative hosts instead (Davies, 2000, 2011; Soler, 2014). Visually, the parasitic eggs in this study were highly mimetic of those of the grey bushchat. This suggests that interactions between the common cuckoo and grey bushchat have progressed to an advanced stage, and that the common cuckoo may lay eggs in other host nests in search of new potential hosts.
2. Possible erroneous parasitism by the common cuckoo. One grey bushchat nest in the same breeding stage was found within 5 m of the nest of the parasitised south rock bunting in this study, which is the main host of the main cuckoo population in the study area. Thus, grey bush chat activity during nesting and egg laying may have already attracted the common cuckoo. During parasitism, the common cuckoo may mistakenly lay its parasitic eggs in the nest of its host's ‘neighbour’ under the pressure of the host's nest defences. The grey bushchat is the dominant species in the study area and has a large population, with its nesting population well distributed (Zhong, 2020). The grey bushchat nests on open bowl‐shaped nests on the ground, similarly to the three bunting species in the present study. And, although many previous studies have suggested that cuckoos select host nests with eggs of phenotypes that match their own for parasitisation (Avilés et al., 2006; Cherry et al., 2007; Honza et al., 2014; Zhang et al., 2023), several recent studies have suggested that cuckoos do not have directional egg laying and the selection process is random instead (Antonov et al., 2012; Spottiswoode & Stevens, 2010; Wang & Liang, 2023; Yang et al., 2010). For example, Yang et al. (2016) found that cuckoos lay eggs indiscriminately in host nests with eggs of various phenotypes, suggesting that cuckoos lay eggs randomly rather than have directional egg laying. Therefore, if there were other ground‐nesting bird species in the vicinity of grey bushchat nests during the egg‐laying period, although they were not the target host, they may be mistakenly parasitised by the common cuckoo.
3. Possible selection of hosts based on the nest site environment and nest type by the common cuckoo. The natal philopatry hypothesis (Brooke & Davies, 1991) and the nest site preference hypothesis (Moksnes & Røskaft, 1995) suggest that the common cuckoo show preference for the nest site environment and nest type rather than a particular host. The grey bushchat shares a similar nest site environment and nest type with all three species of buntings examined. As all three bunting species retain a strong egg rejection ability and the parasitic egg phenotype does not mimic that of the host egg, most eggs parasitised in their nests were rejected.

In summary, this study found low parasitism of all three ground‐nesting buntings by the common cuckoo. However, high levels of rejection of non‐mimetic eggs were observed in all three bunting species. The reason for this may be that they have all been subjected to high parasitic stress and have had a long co‐evolutionary history with a population of cuckoos thus retaining egg recognition ability. However, there was no direct evidence in this study to prove the hypothesis, which needs to be further tested in the future. To the best of our knowledge, this study is the first report on brood parasitism of the yellow‐throated bunting and south rock bunting by the common cuckoo. The common cuckoo of this clade has evolved highly mimetic parasitic eggs and primarily uses the grey bushchat as its main host. These three species of buntings do not have a history of coevolution with the common cuckoo of this clade. The reasons for the parasitism of the yellow‐throated bunting and south rock bunting may be the result of host transfer, chance occurrence, or nest site preference. However, this needs to be further confirmed through future research.

## AUTHOR CONTRIBUTIONS

**Yuhan Zhang:** Investigation (equal); methodology (equal); writing – original draft (equal). **Guo Zhong:** Formal analysis (equal); investigation (equal); methodology (equal). **Guixia Wan:** Investigation (equal); methodology (equal). **Longwu Wang:** Writing – review and editing (equal). **Wei Liang:** Writing – review and editing (equal).

## FUNDING INFORMATION

This work was supported by the National Natural Science Foundation of China (Nos. 32260253 and 31960105 to LW, 31970427 and 32270526 to WL). LW was funded by the Guizhou Natural Science Foundation (No. ZK [2022]‐316), and WL supported by the specific research fund of The Innovation Platform for Academicians of Hainan Province.

## CONFLICT OF INTEREST STATEMENT

The authors declare that they have no competing interests.

## Data Availability

Data in this manuscript are available at the Dryad Digital Repository: https://datadryad.org/stash/share/bgg2j8RjtA69fp2gdIoBc6Uo9VFFvW7X8QGfgMv0vYc.
